# Assisted Gene Flow Yields Physiologically Robust *Acropora palmata* With Novel Allelic and Transcriptomic Profiles

**DOI:** 10.1111/eva.70321

**Published:** 2026-09-23

**Authors:** Erinn M. Muller, Chelsea Petrik, Trinity Conn, C. Cornelia Osborne, Marina Villoch, Abigail S. Clark, Hanna R. Koch, Keri O'Neil, Cody Engelsma, Iliana B. Baums

**Affiliations:** ^1^ Mote Marine Laboratory Sarasota Florida USA; ^2^ Mote Marine Laboratory Summerland Key Florida USA; ^3^ Department of Biology The Pennsylvania State University University Park Pennsylvania USA; ^4^ Brinton Environmental Center Sea Base, Scouting America Summerland Key Florida USA; ^5^ Monroe County BOCC Marathon Florida USA; ^6^ The Florida Aquarium Tampa Florida USA; ^7^ Helmholtz‐Institute for Functional Marine Biodiversity at the University of Oldenburg [HIFMB] Oldenburg Germany; ^8^ Alfred Wegener Institute, Helmholtz‐Centre for Polar and Marine Research [AWI] Bremerhaven Germany; ^9^ Institute for Chemistry and Biology of the Marine Environment (ICBM), School of Mathematics and Science Carl von Ossietzky Universität Oldenburg Oldenburg Germany

**Keywords:** Caribbean, *Durisdinium trenchii*, elkhorn coral, endangered species, genetic diversity, genetic rescue, holobiont, outbreeding, phenotypic response, private alleles

## Abstract

Assisted gene flow (AGF) is a conservation approach that facilitates the spread of alleles and may accelerate the recovery of genetically depauperate populations. The threatened Caribbean coral *Acropora palmata* is approaching regional extinction within the western Atlantic partly due to increasing water temperatures associated with global climate change. Previously, AGF was conducted by crossing gametes collected from three regions (Curaçao—CU, Florida—FL, and Puerto Rico—PR) characterized by contrasting temperature regimes and low gene flow among them. Here, we compared the physiology of these AGF cohorts in comparison to purebred Florida and Curaçao cohorts under ambient and high temperature conditions. Exposure to high temperatures resulted in few physiological changes, likely because the corals hosted the thermally tolerant algal symbiont, *Durusdinium trenchii*. However, the purebred cohorts grew significantly faster than both AGF cohorts. Like the phenotypic responses, gene expression changes in response to heat stress were muted overall. However, AGF cohorts deviated from the expected mid‐parent value across dozens of genes, representing novel gene expression patterns. Importantly, AGF crosses harbored 879 private alleles that were previously not recovered in representative genets from Florida. These findings suggest that corals created using AGF have similar physiology to purebreds, carry novel alleles, and represent novel gene expression patterns that carry important conservation values which could be integrated into the functionally extinct *A. palmata* population in Florida.

## 1 Introduction

Assisted gene flow (AGF) is the intentional translocation of individuals or gametes within a species' range (Aitken and Whitlock 2013) and can be used to conduct several genetic management strategies: genetic rescue, genetic augmentation, and evolutionary rescue (e.g., provenancing). Genetic rescue uses AGF to increase the fitness of small, inbred, or declining populations. Genetic augmentation aims to increase the genetic diversity and adaptive capacity of populations that may not be severely inbred but have low genetic variation, whereas evolutionary rescue facilitates adaptation to rapid environmental shifts, such as climate change, by moving pre‐adapted genotypes (e.g., from warmer to cooler locations). With the increasing rate of extinction (Ceballos et al. 2015) and anthropogenically induced climate change (Masson‐Delmotte et al. 2022), bold interventions such as AGF may be needed to ensure the persistence of species that are unable to endure under current conditions. AGF has already been employed for some forest trees (Morgenstern and Mullin 1990; Rehfeldt et al. 2001; Girardin et al. 2021) and had positive fitness outcomes across diverse animal taxa (Clarke et al. 2024). Indeed, AGF may be a critical tool for preserving species at risk of local extinction, especially species that are at risk of inbreeding depression from recent large population declines. This is because AGF provides recipient populations with genetic diversity from elsewhere in the species' range, and AGF crosses are likely to produce offspring with lower average kinship than local crosses within a genetically depauperate population. Increasing genetic diversity and decreasing average kinship are two critical population management goals that can be achieved via AGF, which helps in preventing inbreeding depression, maintaining beneficial alleles, and promoting adaptive capacity (Aitken and Whitlock 2013).

Coral reefs are experiencing rapid ocean warming, which has led to an increase in the frequency and severity of coral bleaching events and the subsequent mortality of corals worldwide (Hughes, Anderson, Connolly, et al. 2018; Hughes, Kerry, Baird, et al. 2018; Hughes, Kerry, and Simpson 2018). The loss of corals has resulted in increased investments in coral reef conservation initiatives and the development of innovative restoration interventions for preserving species and reef function (van Oppen et al. 2015, 2017; Committee on Interventions to Increase the Resilience of Coral Reefs et al. 2019; Baums et al. 2019). In the eastern Atlantic and Caribbean region, the focus has been on the *Acropora palmata* and *A. cervicornis* , two major reef building species (Pandolfi 2002) that have undergone extensive range‐wide declines from disease outbreaks and bleaching events (Aronson and Precht 2001; Patterson et al. 2002; Williams et al. 2008, 2020). These species are listed as threatened under the US Endangered Species Act (NOAA, C.D. 2014) and critically endangered under the IUCN Red List (Aronson et al. 2008; Green et al. 2008; Williams et al. 2024). Today, there are only 37 living wild *A. palmata* throughout Florida's Coral Reef (Williams et al. 2024) and approximately 150 *Acropora palmata* unique genotypes remaining, which are primarily under human care (Williams et al. 2024; Muller et al. 2026). Furthermore, because many of these genotypes are held as fragments rather than reproductive sized colonies, the effective population size is much smaller. Because of these losses, *A. palmata* is now considered functionally extinct within Florida's Coral Reef (Manzello et al. 2025).

*Acropora palmata* has been targeted in cryopreservation studies (Hagedorn et al. 2012, 2021) because of its severely declining population and inbreeding potential due to the small effective population size. Cryopreservation is an important conservation tool that has proven successful at preserving mammalian embryos (Fahy et al. 1984; Pollard and Leibo 1994; Kasai 1996), fish spermatogonial cells (Hagedorn et al. 2018), and coral larvae (Daly et al. 2018), sperm (Hagedorn et al. 2006, 2012), and microfragments (Powell‐Palm et al. 2023). Since *A. palmata* gametes are only viable for a few hours post‐release, cryopreservation of the sperm has tremendous implications for the propagation and restoration of *A. palmata* . Region‐wide assessments showed there are two genetically distinct populations of *A. palmata* in the Caribbean and northwestern Atlantic with a central, mixed genetic zone around Puerto Rico (Devlin‐Durante and Baums 2017). To assess reproductive compatibility and determine if cryopreservation could be used to facilitate AGF among these geographically separated and genetically isolated populations of *A. palmata* , Hagedorn et al. (2021) collected and cryopreserved sperm from *A. palmata* colonies located in the western Atlantic (Florida, USA), central Caribbean (Puerto Rico), and southern Caribbean (Curaçao). The cryopreserved sperm was then crossed with freshly collected eggs from Curaçao to produce viable offspring, demonstrating that cryopreservation can facilitate AGF among *A. palmata* populations and potentially play an important role in the genetic rescue of the critically endangered *A. palmata* population in Florida. This proof‐of‐concept study was highly successful and produced hundreds of AGF offspring, all of which have been held in isolation within land‐based nurseries since their creation.

Despite the intended benefits of AGF, unintended negative outcomes are possible including outbreeding depression, which is the reduction in fitness resulting from mating genetically distinct populations (Frankham et al. 2011). Potential mechanisms include the loss of locally adapted alleles (Templeton et al. 1986), or disruption of co‐adapted genes at different loci (epistasis) (Edmands 2007). Outbreeding depression is possible between Florida and Curaçao populations insofar as these locations may not be regularly exchanging alleles (Baums et al. 2005, 2006). Puerto Rico, however, has been identified as an area of mixing between the western and eastern Caribbean regions (Baums et al. 2005, 2006). Even though outbreeding depression has never been directly tested in stony corals, risks associated with AGF are predicted to be low (Baums et al. 2019), compared to its potential benefits in rapidly declining local populations that are failing to reproduce sexually (Ralls et al. 2018), such as the functionally extinct *A. palmata* population in Florida.

Another consideration of performing AGF with stony corals is that these species possess an obligate, mutually beneficial symbiosis with unicellular algae (Family: Symbiodiniaceae) that can influence host physiology and stress responses (Mieog et al. 2009; Howells et al. 2012). Therefore, the phenotype of an adult coral is the result of the algal endosymbiont and host together (Parkinson and Baums 2014). This coral‐algal symbiosis is especially important when it comes to thermal tolerance and coral bleaching. Under prolonged temperature extremes, the symbiosis breaks down resulting in endosymbiont expulsion, coral ‘bleaching’, starvation, and eventually death if the stress does not subside and symbiosis is re‐established. However, symbiont species differ in their physiological optima and influence the stress phenotype of their host under changing environmental conditions (Little et al. 2004; Cunning et al. 2015). *A. palmata* associates with *Symbiodinium ‘fitti’* (nominem nudum) and adults only rarely host other symbionts such as *Durisdinium trenchii* or *Cladocopium* sp. (Baums et al. 2014). Juvenile *A. palmata* are more flexible and sometimes host *D. trenchii*, which could be retained for years in adults (Elder et al. 2023).

Although evolutionary rescue, which facilitates adaptation to rapid environmental shifts, such as climate change, was not the original intention of the Hagedorn et al. (2021) effort, *A. palmata* populations in Florida will need to withstand extreme temperature anomalies to survive (USGCRP 2023). Additionally, the current depauperate status of *A. palmata* in Florida suggests AGF may provide substantial benefits to the existing population. Therefore, to evaluate the conservation potential of AGF for *A. palmata* in Florida, we conducted a thermal stress experiment comparing F1 offspring from two AGF cohorts (Curaçao × Florida and Curaçao × Puerto Rico) against two purebred cohorts (Florida × Florida and Curaçao × Curaçao) created from the efforts outlined in Hagedorn et al. (2021). Coral fragments from all four cohorts were reared under either ambient (28°C) or elevated (31°C) temperature conditions for 8 weeks—ultimately exposing the high temperature treatment corals to 11.2 degree heating weeks (based on Florida's mean monthly maximum, Liu et al. 2003). Throughout and at the conclusion of the experiment, we assessed a comprehensive suite of physiological and genomic responses, including coral metabolism (net photosynthesis and respiration rates, daily P:R ratios), growth, algal symbiont photophysiology, whole‐transcriptome gene expression via RNASeq, and host population genetic structure via single nucleotide polymorphism (SNP) genotyping. Together, these measurements allowed us to determine physiological differences among cohorts, assess if AGF crosses confer physiological resilience to heat stress relative to purebred cohorts, and whether AGF offspring carry novel genetic and transcriptomic variation of conservation value.

## 2 Materials and Methods

### 2.1 Study Site, Species, and Cohorts

The present study was conducted at Mote Marine Laboratory's Elizabeth Moore International Center for Coral Reef Research & Restoration facility (24°39′41″ N, 81° 27′ 16″ W, Summerland Key, Florida, USA). The *Acropora palmata* corals used in this study originated from Hagedorn et al. (2021), which were created in 2018 and settled and maintained in Mote's land‐based nursery or The Florida Aquarium. Only corals from Mote's land‐based nursery were used in the present study and represent four different *A. palmata* cohorts (crosses): (i) a batch cross of fresh eggs and sperm from the Upper Florida Keys (FL × FL; five dams and sires); (ii) a batch cross of fresh eggs and sperm from Curaçao (CU × CU; five dams and 4 or 5 sires depending on the night); (iii) a cross between fresh eggs from Curaçao and cryopreserved sperm from Florida (CU × FL; five dams and two sires); and (iv) a cross between fresh eggs from Curaçao and cryopreserved sperm from Puerto Rico (CU × PR; five dams and five sires). The same five dams were used for all cohorts that utilized Curacao eggs. Further details on the cohort creation can be found in Hagedorn et al. (2021). The CU × FL and CU × PR crosses represent the cohorts created through assisted gene flow and will be referred to as ‘AGF’ corals and/or ‘AGF’ cohorts, while the other two represent ‘purebred’ cohorts. For population‐level analyses, we further included existing genotyping data from randomly selected wild Florida Keys (*n* = 30), Curaçao (*n* = 30) and western Puerto Rico (*n* = 27) genets. These genets were genotyped using the same methods as the experimental crosses and are publicly available in the STAG database (coralsnp.science.psu.edu/galaxy). Genotyping methods are described below.

### 2.2 Experimental Set‐Up

The thermal stress experiment was carried out within Mote's Climate and Acidification Ocean Simulator (CAOS) system from 15 June to 2 September 2020. A total of 50 putatively different genotypes, which were approximately two years old, from each of the four cohorts reared within Mote's land‐based coral nursery were initially fragmented in half. Putative genotypes were haphazardly selected from the available cohorts. Colonies were fragmented to 1–2 cm^2^ in size using a band saw (C‐40 diamond Band saw, Gryphon) and mounted onto flat ceramic plugs (1 ¼″ diameter, Boston Aqua Farms) with cyanoacrylate gel. The fragmented corals were given two weeks to recover and acclimate within the CAOS system before the onset of the experiment. One of each replicate per cohort (*n* = 50) was placed within the ambient temperature treatment (control) and the other replicate was placed within the high temperature treatment (experimental). This created a total of 200 fragments per treatment (50 replicates per cohort, four cohorts per treatment).

**FIGURE 1:**
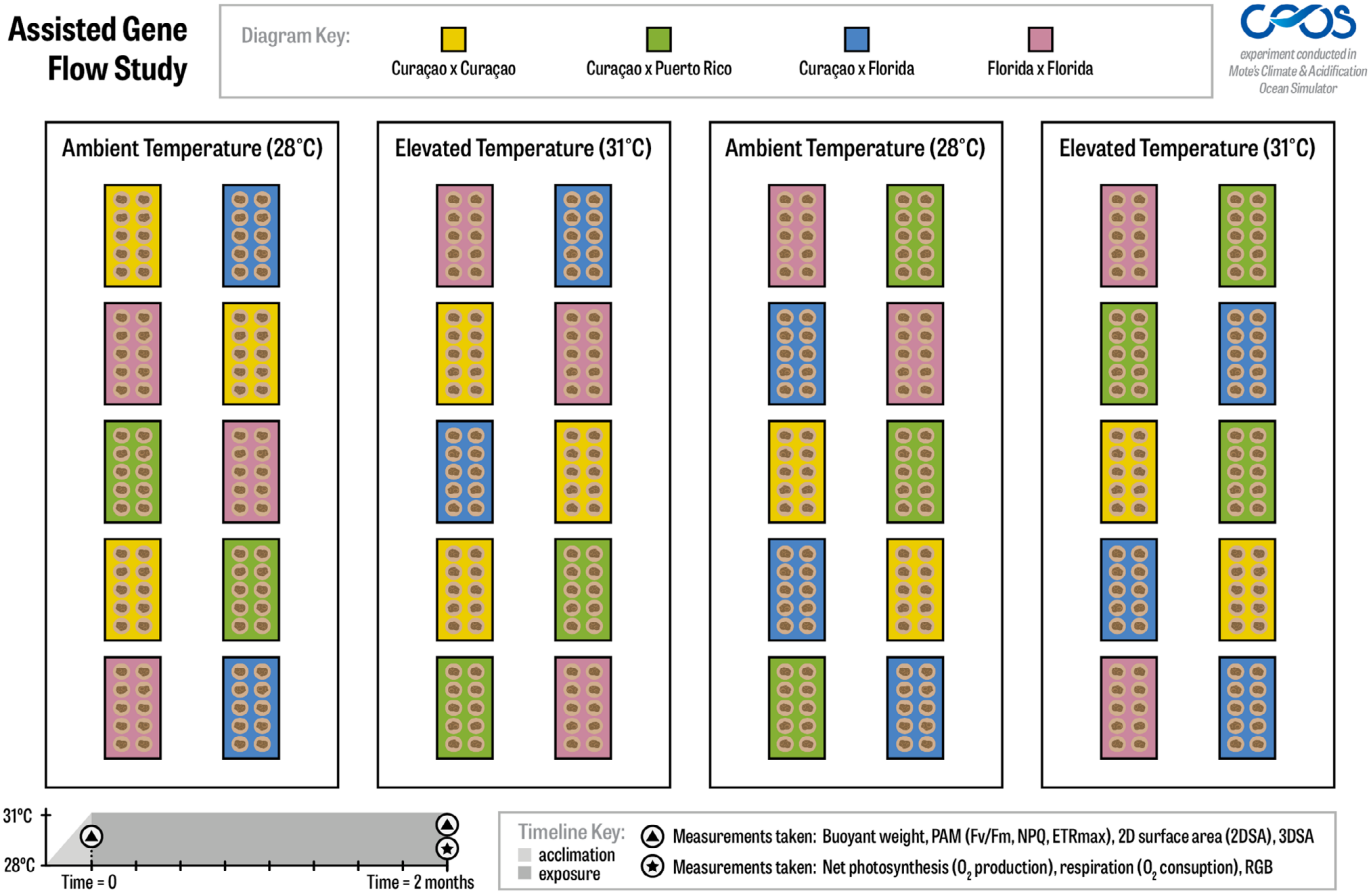
Schematic representation of the experimental design used within the present study.

To assess cohort‐level responses to heat stress, coral fragments were subjected to one of two temperature treatments, ambient (28°C) or elevated (31°C), for 2 months. Ambient temperature was modeled after the mean temperature for June curated from temperature loggers at Looe Key Reef, which has recorded in situ temperature at 6 m for the past 15 years. The temperature of 31°C was selected for the elevated temperature treatment as 30.5°C is estimated to be the bleaching threshold reported for Acroporid corals in the Florida Keys (Manzello et al. 2007; Williams et al. 2017). Furthermore, eight weeks at 31°C is equivalent to 11.2 DHW in Florida (mean monthly maximum (MMM) = 29.6°C), 19.2 DHW in Puerto Rico (MMM = 28.6°C), and 24 DHW for Curacao (MMM = 28°C). Values exceeding eight DHW exceed thresholds identified by NOAA that would be expected to cause near‐total bleaching and substantial die‐off in most coral species. On 8 June 2020, mounted corals were placed within their respective 20 L flow‐through glass tanks (16.25″ × 8.375″ × 10.5″) fitted with a transparent Plexiglas lid and submersible pumps (120 GPH; Dwyer). Forty tanks were held within four shallow fiberglass flow‐through mesocosms (‘raceways’; 100″ × 40″ × 12″) under an overhead shaded structure (~75% light reduction) outside. Each tank held 9–10 replicates from a single cohort and each cohort consisted of five separate tanks per treatment. Temperature was regulated in the raceway and maintained at either ambient (28°C) or high (31°C) conditions. On 25 June 2020 (Figure 1), after preliminary physiological measurements were performed, temperatures were manually raised in the water baths containing respective high temperature treated experimental tanks at a rate of +0.5°C per day until the target temperature of 31°C was met. Temperatures were controlled by a digital temperature controller (TSW‐250, Dwyer) with inline sensors and heat exchangers via a central heater (Lochinvar‐Knight, 286 MBH Hi‐Efficiency Boiler) or chiller (15 Ton Air Chilled Cooler). Once at target temperature conditions, the temperatures were held constant (deadband ±0.2°C) for 8 weeks.

Water quality parameters including temperature (°C), pH_NBS_ (NBS: National Bureau of Standards), dissolved oxygen (DO; mg/L), and salinity (ppt) were measured daily for each experimental tank using a multi‐parameter handheld device containing a conductivity/temperature sensor, galvanic DO sensor, and a pH sensor (YSI professional series sensors). The pH sensor was calibrated daily using NBS scale standard buffers (4, 7, and 10). Corals were fed once a week near dusk (100–200 μm, Golden Pearls, Aquatic Foods Inc.). Light levels (photosynthetically active radiation, PAR) were recorded with a planar underwater quantum sensor (LI‐1500 and LI‐192, Li‐Cor) near maximum irradiance (approx. 13:00 GMT −5) every 3 days.

### 2.3 Growth Rates

Coral fragment calcification rates were measured using the buoyant weight method (adapted from Spencer Davies 1989). Corals were weighed prior to exposure and again after 8 weeks of exposure by placing the coral on a suspended egg‐crate (diffuse styrene lighting panel) platform connected to a monofilament line below a precision balance where the stabilized weight was recorded. One coral per tank was selected haphazardly to be weighed in duplicate to ensure accuracy of the scales; the difference between the duplicate weights was low (< 0.008 g) suggesting dependable measurements. Dry weight was calculated from methods described by Jokiel et al. (1978) and the change of weight was characterized, as a relative unit, by subtracting the initial dry weight from the final dry weight and adjusted per initial two‐dimensional (2D) surface area per day (cm^2^ day^−1^). During weighing periods, overhead photographs were taken of each coral replicate to derive 2D surface area (cm^2^) where image analysis was performed in ImageJ (National Institutes of Health). Change of 2D surface area was calculated from the difference between the final size and the initial size and standardized per day (cm^2^ day^−1^).

Three‐dimensional (3D) surface area for 20 replicates per cohort per temperature treatment was measured using a structured‐light 3D scanner. The subset of corals was selected using a random number generator (i.e., RAND() function) with fixed values to prevent re‐randomization in Microsoft Excel. Coral images were taken using a HDI Compact scanner (C210 Polyga, Burnaby, BC Canada) on a rotating plate following the methods of Koch et al. (2021). Images were captured every 30° in one rotation, from one angle, to produce 12 meshes. The High Dynamic range (HDR) setting was used as dual exposure was necessary to appropriately capture and differentiate between surfaces of the ceramic plug (coral mount) and actual coral tissue. The 3D scanner was calibrated prior to measurements using a 5 mm calibration board. For the analyses, meshes were uploaded to the Flexscan3D software to be reconstructed manually, and finalization of the model was performed. Mesh editing (i.e., hole filling and smooth merge functions) was applied where necessary. Once the 3D model was finalized, the model was trimmed to exclude the plug. Once editing and trimming were completed, the model was measured for remaining surface area (mm^2^) as described by Koch et al. (2021). Change in 3D surface area was calculated from the difference between the final size and the initial size (mm^2^).

### 2.4 Respirometry

Twelve replicate corals per cohort per temperature treatment were measured for oxygen production and oxygen consumption at the end of the 8‐week experiment. The subset of corals was selected using a random number generator (i.e., RAND() function) with fixed values to prevent re‐randomization in Microsoft Excel. Net photosynthesis (P_net_) and respiration (R) rates were measured with a fiber‐optic oxygen meter (Firesting O_2_, Pyro Science GmbH, Aachen, Germany) connected to a computer with Pyro Oxygen Logger software. An oxygen sensor was placed in each incubation chamber (300 mL) coupled with magnetic stir bars for adequate mixing. The oxygen sensors were calibrated prior to measurements in 100% air saturated water. Chambers were surrounded by a recirculating jacket at the appropriate treatment temperature (28°C or 31°C). Chambers were filled with seawater from the corals' respective tank for every measurement. Each chamber contained one replicate coral. One chamber per each incubation period contained seawater only as an empty control, or ‘blank’, to correct rates for background microbial respiration. A standard fluorescent light strip was used as a lighting source and placed overhead at a fixed distance from the chambers. Photosynthesis rates (oxygen production) were measured at approximately 52 ± 11 μmol photons m^−2^ s^−1^ for 60 min. Respiration (oxygen consumption) rates were measured in complete darkness for 60 min. Rates were normalized per two‐dimensional surface area (cm^2^) of the coral fragments taken at the final time point. Daily P:R was calculated following the equation in Krueger (2019), where daily P:R was the P_gross_ multiplied with the number of daylight hours (12 h) and then divided by the product of 24 h times the hourly respiration value.

### 2.5 Algal Symbiont Traits

An imaging pulse amplitude modulated (I‐PAM) M‐Series fluorometer (Heinz Walz GmbH, Effeltrich, Germany) was used to measure photosynthetic efficiency of the algal endosymbiont, the maximum potential quantum efficiency of photosystem II (*F*_v_/*F*_m_). Instrument settings were as follows: light intensity = 6, saturation pulse intensity = 8, gain = 1–3. Measurements were taken at a fixed distance from the coral tissue in the center of the fragment. Corals were placed in complete darkness for 30 min prior to each measurement. A weak measuring light (< 1 μmol quanta m^−2^ s^−1^) was used to obtain the minimum fluorescence (*F*_o_) followed by a saturating pulse (3000 μmol quanta m^−2^ s^−1^) to measure the maximum fluorescence (*F*_m_), which were used to calculate the ratio of variable fluorescence to maximal fluorescence (*F*_v_/*F*_m_). *F*_v_/*F*_m_ was measured for all fragments prior to administration of experimental treatments and after 8 weeks. After *F*_v_/*F*_m_ measurement, light intensities ranging from 0 to 1280 μmol quanta m^−2^ s^−1^ on intervals of 20 s were emitted on the sample to produce rapid light curves (RLCs). ETR_max_ was determined by multiplying the effective quantum yield (measured during RLCs) by photo flux density (400–700 nm) (Platt et al. 1980). The maximum level of non‐photochemical quenching within the light curve (NPQ_max_) was calculated as (*F*_m_ − *F*_m′_)/*F*_m′_; where quenched *F*_m_ is called *F*_m′_ (Krause and Weis 1991; Ruban 2016).

Overhead two‐dimensional photographs were taken of each fragment at three time points (pre‐exposure, 1 month post exposure, 2 months post exposure) to measure change in tissue surface area, as previously described, and change in pigmentation. Photographs were taken with a digital camera (Olympus TG‐6) held at a fixed distance above the corals where a metric ruler and white/black color calibration card were placed within frame on the same plane as the coral fragment. Camera settings for all photographs were pre‐fixed and included an aperture speed of 1/60s, ISO = 120–160, *F* = 2.8, with the flash off. Color analysis was completed at the final time point based on the red, green, and blue (RGB) color model as described by Winters et al. (Winters et al. 2009). RGB channel color intensity was based on the 0–255 scale. Normalization of image color was performed prior to a histogram analysis for each contributing color in GIMP software (version 2.10). All phenotype data collected are provided in Table S1.

### 2.6 Tissue Sampling for Gene Expression and Cohort Genomic Analyses

From 11 to 13 June 2020, all corals (*n* = 400) were sampled to determine baseline gene expression levels before heat stress exposure. Using a sterile precision knife, two to five polyps were carefully removed from each coral and immediately placed in a 2 mL cryogenic tube containing 1.5 mL RNA*later* (Invitrogen), followed by storage in a 4°C refrigerator for 24 h. After 24 h, the samples were transferred to a −80°C freezer and stored until further analysis. Once the physiological measurements were completed (at the 8‐week time point), all remaining corals were resampled for gene expression analysis (*n* = 364) and for population genomic analysis (*n* = 400) post‐heat stress. Thirty‐six corals had significant tissue loss prior to the end of the experiment (5 September 2020) and were therefore sampled before the conclusion of the study. An additional 36 corals suffered complete mortality during the experiment and were consequently not sampled. In total, 762 samples were taken for gene expression and population genomic analyses with baseline sampling distributed evenly at ~50 corals per cohort per temperature treatment; post‐stress sampling included CU × CU (Ambient: *n* = 37, Elevated: *n* = 48), CU × FL (Ambient: *n* = 50, Elevated: *n* = 49), CU × PR (Ambient: *n* = 41, Elevated: *n* = 40), and FL × FL (Ambient: *n* = 48, Elevated: *n* = 49). After samples were collected for gene expression analysis, all remaining tissue was flash‐frozen and stored at −80°C. Samples were transported to Pennsylvania State University and stored at −80°C until further analysis.

### 2.7 DNA Extraction and Genotyping

Samples were collected using a razor blade from all 200 genets included in the experiment. Samples were extracted using the QIAGEN DNeasy Blood and Tissue kit following published protocols (Kitchen et al. 2020). DNA was quantified at ThermoFisher via picogreen, and DNA quantities ranged from 1.71 to 79.38 ng μL^−1^. DNA was sent to ThermoFisher for genotyping using the Applied Biosystems Axiom Coral Genotyping Array—550,962 following Kitchen et al. (2020). Quality control analyses were performed by Thermofisher. 19,969 probes on the array were designed to resolve the population genomic structure of *A. palmata* and *A. cervicornis*. Subsequent analyses were performed using this set. After removing all samples that failed or were not included in the physiological measurements, 191 samples remained for subsequent analyses.

### 2.8 Symbiont Typing

Of the 4021 probes designed for the Symbiodineaceae hosted by the coral, 54 probes were designed specifically to resolve genera (Kitchen et al. 2020). The raw signal intensities of these probes were exported using the Axiom Analysis Suite and using methods described in Kitchen et al. (2020), a Linear Discriminant Analysis (LDA) was used to determine the genera of Symbiodiniaceae was hosted by each sample of *Acropora palmata* .

### 2.9 Genetic Diversity Analyses

To quantify genetic differentiation across all four AGF cohorts and three wild populations, a Principal Component Analysis (PCA) was performed on genotyped AGF (*n* = 28–60) samples using all 19,965 SNPs (single nucleotide polymorphisms). Next, data from wild genets from the Florida Keys (*n* = 21), Curaçao (*n* = 27), and Western Puerto Rico (*n* = 35) were randomly selected from the Acropora StagDB (www.coral.snp.psu.edu) and added to the AGF cohorts to quantify how genetically similar the AGF cohorts were to their source populations (Figure 6b).

Pairwise kinship coefficients were calculated for all corals using the KING robust method (Manichaikul et al. 2010). The experimental corals were bred from a small pool of parents. Therefore, we expected that experimental corals would be more closely related to each other than a comparable number of wild colonies from any reef. This is of interest because closely related genets are more likely to respond to stressors similarly than unrelated genets (Dixon et al. 2015; Howells et al. 2021). An ANOVA was performed to calculate the differences in kinship within and between cohorts. To assess whether these cohorts represented similar pools of genetic diversity and were representative of their wild source populations, multiple metrics of allelic diversity were quantified in the cohorts and representative samples of the wild populations. First, allelic richness was quantified across all markers using the package PopGenReport (Adamack and Gruber 2014) in R (R Core Team), and an ANOVA was performed to compare allelic richness across the genome among all cohorts. Second, private alleles, alleles found exclusively in one specific population or geographic area, between the three parent populations (CU, FL, PR) were identified using the package Poppr (Kamvar et al. 2014) from the wild genets selected from the Acropora StagDB previously described. Discovered private alleles had a frequency of 0.01 to 1.0 in the wild populations. We then identified and summed which of these alleles were carried by the AGF cohorts and not found in their respective natal population. Finally, observed heterozygosity, expected heterozygosity, and *F*_IS_ (inbreeding coefficient) were calculated for each cohort using the R package dartR (Gruber et al. 2018). All statistical analyses were performed using R v.4.0.2.

### 2.10 Genotype–Phenotype Association

To test if there were any genotyped SNPs associated with the observed differences across cohorts, a genome‐wide association study (GWAS) was performed using plink2 (Chang et al. 2015). The association test was performed on 5 traits with large enough sample sizes: 2D Surface Area, Buoyant Weight, *F*_v_/*F*_m_, NPQ_max_, and ETR_max_. Of 19,965 SNPs, 6445 polymorphic SNPs were retained for analysis. 377 of those SNPs were removed due to a minor allele frequency below 0.01. A KING kinship cutoff of 0.2 was established to prune any closely related recruits. To account for population structure, the top 10 principal components of the population structure were extracted using plink2 and input into the linear model as a covariate. Traits were analyzed as the change in each trait between the first sampling time‐point and the final sampling time‐point in all elevated‐temperature samples. A Bonferroni correction was applied to determine alleles that showed a significant association with the tested phenotypes.

### 2.11 RNA Extraction and Differential Gene Expression Analysis

Total RNA was extracted from all 762 samples for gene expression analyses. For each sample, 2–3 polyps were homogenized in 1 mL TRIzol reagent (Ambion, Life Technology, USA) before centrifugation with 200 μLl chloroform for 15 min at 12,000*g*, 4°C. The aqueous phase was removed and cleaned using Qiagen RNeasy Mini Kit, as per manufacturer's protocol with an additional on‐column DNase treatment (RNase‐Free DNase Set, Qiagen). The eluted RNA (in RNAse‐free water) was twice passed through the spin column to maximize final concentration. Concentration and purity (A260/280, 260/230) were analyzed via NanoDrop ND‐1000 spectrometer and quality assessed (RIN > 7) with Agilent 2100 Bioanalyzer (Agilent Technologies). Samples were submitted to Admera Health (New Jersey, USA) for removal of salt and 2 × 150 bp paired‐end sequencing using the NEBNext Ultra II Nondirectional Library Prep Kit with polyA selection (New England Biolabs Inc.) Illumina universal adapters and reads below PHRED of 23 were trimmed using Cutadapt (v 3.5 Martin 2011). Filtered reads were mapped to the *Acropora palmata* genome (v3.1, Kitchen et al. 2020, unpublished) using *STAR* (v2.7.0a) with read count data generated by the ‐quantMode GeneCount parameter. Following sequencing and alignment quality control, 380 samples were retained for DESeq2 input.

Differentially expressed genes (DEGs) were identified with the R package *DESeq2* (Love et al. 2014). Genes with fewer than 10 counts across any gene were discarded, leaving a total of 17,983 genes in the dataset. We first fit the model Expression ~ Cohort on the baseline expression data to identify any cohort‐specific transcriptomic differences that may differentiate the populations under ambient conditions. To then assess the transcriptomic response to elevated temperatures, the model Expression~Cohort*Treatment was used and pairwise contrasts were extracted to analyze (1) the cohort‐specific gene expression under heat stress and (2) the differential response between cohorts to heat stress. Genes identified as differentially expressed were corrected for multiple testing using Benjamini and Hochberg false discovery rate (FDR) (Benjamini and Hochberg 1995). Over‐representation tests of gene ontology (GO) terms were performed using the package *clusterProfiler* (Yu et al. 2012) using a custom GO database for the *Acropora palmata* genome created with the *AnnotationForge* package (Carlson et al. 2024) courtesy of Kitchen et al., in prep. Using the variance stable transformation (*vst*) counts as input, the ‘plotPCA’ function from *DESeq2* was used for PCA analyses to explore cohort, genotype, and treatment effects as well as identify other potential sources of variation such as tank effects and differentiating physiological measurements identified in this study. Tank, runway, and PAR were all included in initial models but were not found to be significant factors explaining clustering of the cohorts and were thus excluded from the final model.

In addition, to investigate baseline expression deviation of the AGF crosses from pure‐bred crosses, the AGF‐cross samples of CU × FL were compared against the parental samples of FL × FL and CU × CU, fitting the model ‘expression ~group’. For this analysis, we used median expression values from the two replicate fragments of each genet in the pre‐exposure ambient and pre‐exposure heat‐stress treatments. Expression in the AGF crosses was then categorized as either greater than both parent crosses or below both parent crosses. Using the command rlog() in DESeq2, gene counts were normalized and log‐transformed before use in PCA and heatmaps.

### 2.12 Statistical Analysis of Phenotype Data

A factorial permutational multivariate analysis of variance (PERMANOVA, adonis2 function in the R package ‘vegan’ (V 2.5–7 Oksanen 2020)) was conducted to determine whether there were significant differences in the phenotypes among cohorts, between temperature treatments, and the interaction of the two variables. Because there were tanks nested within cohorts and treatment in the experimental design and permutational analyses are restricted by nesting within the model, values were averaged within each tank, and the tank was utilized as the experimental unit for this analysis. To visualize the data, principal components analyses (PCA; function prcomp in R base program) were applied to the physiological response parameters among the four cohorts and between the two temperature treatments. Traits that were significantly correlated were removed from the PCA. P:R was removed as it was correlated with P_net_ and R. Additionally, ETR_max_, *F*_v_/*F*_m_, and NPQmax were all correlated, so only *F*_v_/*F*_m_ was retained in the multivariate analysis. Data was normalized by using a log transformation after adding a constant integer of 50 to ensure all values were positive prior to the PERMANOVA and PCA applications to create comparable units (Data provided in Table S2).

To determine the effect of temperature treatment, cohort, and the interaction of the two variables, a linear mixed effect model using the R package ‘lme4’ (Bates et al. 2015) for the core fitting model and ‘lmerTest’ (Kuznetsova et al. 2017) for quantification of the *p* values were applied to each dependent variable. Tanks were included as a random value within the model. Normality was evaluated using a Shapiro–Wilk test and homoscedasticity of variances was assessed using Levene's test (LeveneTest function, car package, Fox and Weisberg 2018). When significant differences were detected among the cohorts, a post hoc estimated marginal means test was conducted using the R package ‘emmeans’ (Lenth and Piaskowski 2025).

## 3 Results

### 3.1 Exposure Conditions

The water quality conditions within the experimental tanks remained consistent for the 8 weeks the temperature exposure experiment was conducted (Table 1). Salinity was maintained within a range of 37.4 to 37.5 ppt, dissolved oxygen from 5.81 to 6.00 mg/L, pH_NBS_ from 8.00 to 8.02, and light levels from 410 to 426 μmol photons m^2^ s^−1^. The temperature target for the control conditions was 28°C and the high temperature treatment target of 31°C was met; the experimental tanks were held at 28.0°C ± 0.01°C (mean ± SE) in the control temperature treatment tanks and held at 31.0°C ± 0.01°C in the high temperature treatment tanks across the 8‐week exposure period.

**TABLE 1:** Average (±SE) water quality conditions (temperature, salinity, pH_NBS_, dissolved oxygen, and light) measured in experimental tanks containing the four cohorts held at ambient and high temperature treatments across the exposure period from 1 July 2020 to 4 September 2020.

Temperature treatment	Temperature (°C)	Salinity (ppt)	pH (NBS)	Dissolved oxygen (mg/L)	Light (PAR)
Ambient	28.0 ± 0.01	37.4 ± 0.04	8.02 ± 0.01	6.0 ± 0.0	410 ± 10
High	31.0 ± 0.01	37.5 ± 0.04	8.0 ± 0.002	5.8 ± 0.02	426 ± 9

### 3.2 Physiological Response

**FIGURE 2:**
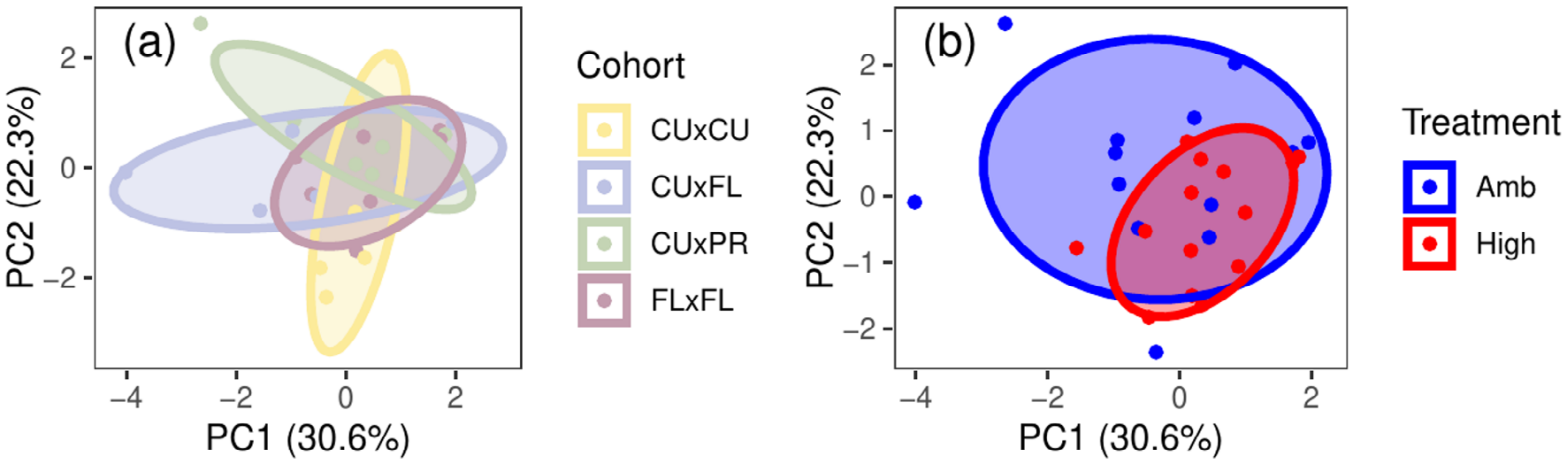
Distance biplots resulting from the principal component analyses (PCA). (a) The multivariate physiological response of the four cohorts of *Acropora palmata* . (b) The multivariate physiological response of the four *Acropora palmata* cohorts under ambient (Amb) or high temperature conditions (High). Physiological traits included were 2D surface area growth (2DSA), 3D surface area growth (3DSA), Calcification rates (BW), Respiration rates (R), Net Photosynthesis rates (P_net_), and Photochemical efficiency (*F*_v_/*F*_m_).

There were no significant differences among cohorts (PERMANOVA: *F*_(3, 19)_ = 1.73, *p* = 0.200) between treatments (PERMANOVA: *F*_(1, 19)_ = 1.83, *p* = 0.198), or the interaction of the two variables (PERMANOVA: *F*_(3, 19)_ = 0.59, *p* = 0.663) when the physiological data were analyzed using a multivariate approach. The PCA visualization also dispalys the overall similarity among the cohorts (Figure 2a) as well as between temperature treatments (Figure 2b) within ordination space.

### 3.3 Growth Metrics (2DSA, 3DSA, BW)

**FIGURE 3:**
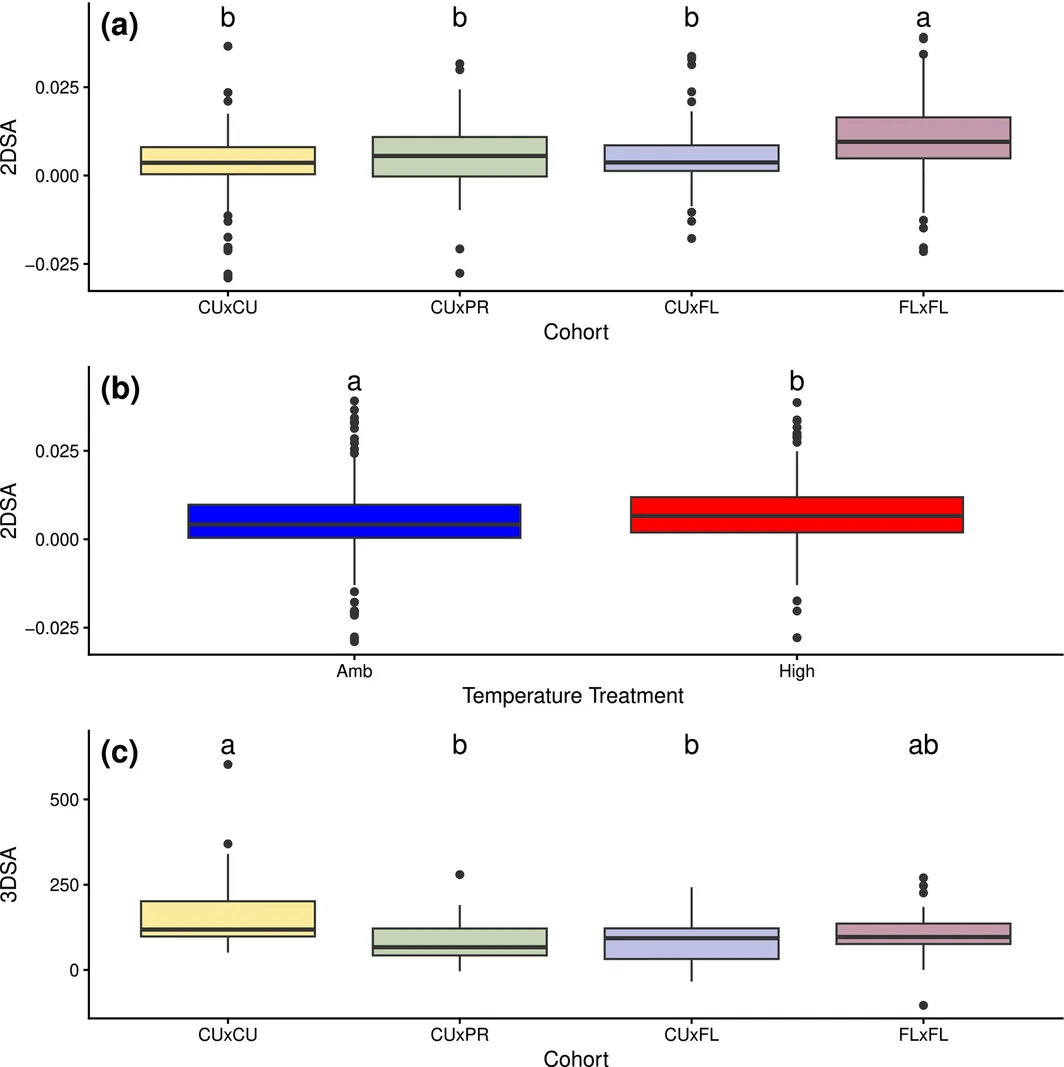
Physiological metrics showed significant differences among cohorts or among temperature treatments. (a) Two‐dimensional surface area (2DSA) of the four different *Acropora palmata* cohorts assessed. (b) The 2DSA of all the cohorts combined under either ambient or high temperature conditions. (c) The three‐dimensional surface area (3DSA) of the four different *A. palmata* cohorts assessed. Horizontal lines represent the medians of each metric, the boxes represent the interquartile range, and the whiskers represent 1.5 times the interquartile range. The dots represent outliers in the dataset. Letters above each metric represent those that are statistically different from others within the comparison.

There were significant differences in growth measured as 2DSA among cohorts (*F*_(1, 32.04)_ = 4.66, *p* = 0.039) and between temperature treatments (*F*_(3, 32.02)_ = 6.54, *p* = 0.001), but no interaction was detected (*F*_(3, 32.02)_ = 0.30, *p* = 0.827). The 'post doc' analysis showed that the FL × FL purebred cohort had significantly higher two‐dimensional growth than all other cohorts (FL × FL vs. CU × CU: *p* = 0.001; FL × FL vs. CU × FL: *p* = 0.037; FL × FL vs. CU × PR: *p* = 0.042; Figure 3a). Visualization of the temperature treatments shows that corals within high temperature treatments also showed significantly more two‐dimensional growth compared with those within ambient conditions (Figure 3b). The growth rates measured as the final 3DSA showed that there were significant differences among cohorts (*F*_(3, 29.11)_ = 3.87, *p* = 0.019; Figure 3c) with the CU × CU cohort having significantly faster three‐dimensional growth compared with the CU × FL AGF cohort (*p* = 0.020) and the CU × PR AGF cohort (*p* = 0.047), but not when compared with the FL × FL cohort (*p* = 0.142). There were no differences in 3DSA measured between temperature treatments (*F*_(1, 29.27)_ = 2.82, *p* = 0.104) or as an interaction between cohort and temperature treatments (*F*_(3, 29.11)_ = 0.15, *p* = 0.928). There were no significant differences in calcification rate (measured as BW) detected among the cohorts (*F*_(3, 32.96)_ = 2.52, *p* = 0.075), between the temperature treatment (*F*_(1, 32.98)_ = 0.02, *p* = 0.880), or with the interaction of the two variables (*F*_(3, 32.96)_ = 0.23, *p* = 0.873) suggesting that all corals accreted a similar amount of skeleton regardless of cohort or temperature conditions.

### 3.4 Respirometry and Algal Symbiont Traits (P_net_, R, Daily P:R, *F*_v_/*F*_m_, ETR_max_ , NPQ_max_ , RGB)

There were no significant differences detected among cohorts (P_net_: *F*_(3, 19.45)_ = 2.01, *p* = 0.145, R: *F*_(3, 19.69)_ = 0.79, *p* = 0.514, daily P:R: *F*_(3, 11.15)_ = 0.42, *p* = 0.741), between temperature conditions (P_net_: *F*_(1, 19.46)_ = 1.48, *p* = 0.239,  R: *F*_(1, 19.71)_ = 0.80, *p* = 0.383, daily P:R: *F*_(1, 11.15)_ = 0.01, *p* = 0.920), or the interaction of cohort and temperature treatment (P_net_: *F*_(3, 19.45)_ = 0.32, *p* = 0.813, R: *F*_(3, 19.69)_ = 0.83, *p* = 0.495, daily P:R: *F*_(3, 11.15)_ = 0.57, *p* = 0.643) for any of the respirometry metrics measured in this study. Similarly, when analyzing the algal symbiont traits there were no significant differences detected among cohorts (*F*_v_/*F*_m_: *F*_(3, 32.10)_ = 2.59, *p* = 0.070; ETR_max_: *F*_(3, 31.75)_ = 2.07, *p* = 0.124; NPQ_max_: *F*_(3, 31.20)_ = 1.76, *p* = 0.175; RGB: *F*_(3, 32.53)_ = 0.61, *p* = 0.613), between temperature treatments (*F*_v_/*F*_m_: *F*_(1, 32.10)_ = 3.03, *p* = 0.091; ETR_max_: *F*_(1, 31.76)_ = 0.61, *p* = 0.441; NPQ_max_: *F*_(1, 31.20)_ = 2.91, *p* = 0.098; RGB: *F*_(1, 32.53)_ = 0.69, *p* = 0.411), or the interaction of cohort and temperature treatment (*F*_v_/*F*_m_: *F*_(3, 32.10)_ = 0.94, *p* = 0.435; ETR_max_: *F*_(3, 31.75)_ = 0.52, *p* = 0.673; NPQ_max_: *F*_(3, 31.20)_ = 1.09, *p* = 0.368; RGB: *F*_(3, 32.53)_ = 0.48, *p* = 0.696).

### 3.5 Gene Expression Results

#### 3.5.1 Baseline Transcriptomic Comparisons

**FIGURE 4:**
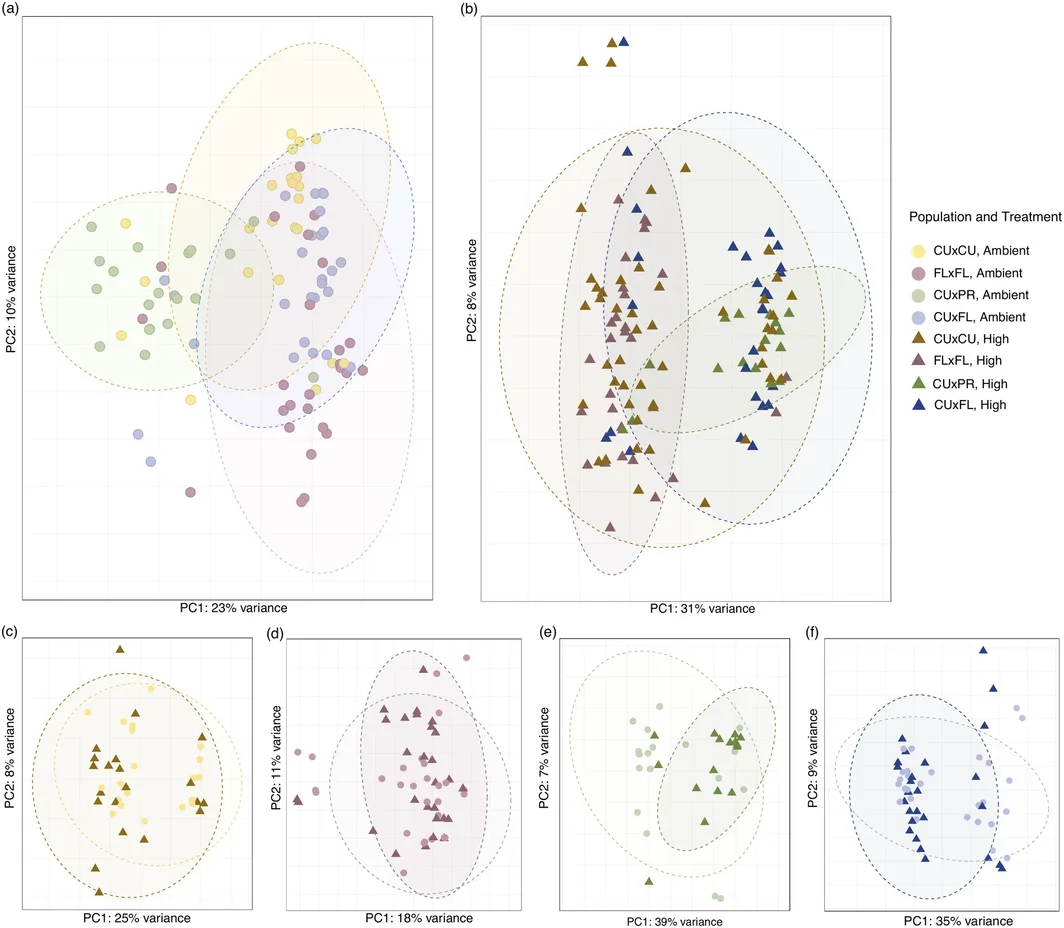
Principal Component Analysis (PCA) of gene count data across cohorts and temperature treatment (ambient temperature samples denoted by lighter colored circles and elevated temperature samples shown as dark‐shade triangles). Baseline gene expression profiles of the four cohorts (a) primarily separates the Curaçao × Puerto Rico (CU × PR, green) AGF crosses from the remaining crosses, explaining 23% of the variance between samples along Principal Component 1 (PC1), while 10% of variance explained by PC2 appears to be driven by the transcriptomic divergence between the non‐AGF Curaçao × Curaçao (CU × CU, yellow) and Florida × Florida (FL × FL, pink) genets. After elevated temperature exposure, temperature treatment did not drive population differentiation, with the four cohorts clustering indiscriminately together (b); none of the variables tested explained the 31% of variance between populations captured along PC1. Neither CU × CU (c) nor FL × FL (d) cohorts showed a significant thermal stress response, only differentially expressing 34 and 20 genes, respectively. AGF cohort CU × PR (e) was more affected by the temperature change, with 175 DEGs explaining 39% of variance on PC1. Curaçao × Florida (CU × FL, blue), (f) had 81 DEGs in response to thermal stress, though the 35% of variation explained by PC1 is not driven by this. Clustering patterns seen in the CU × CU and CU × FL are likely driven by genet‐specific variation rather than treatment response.

Analysis of transcript abundance before temperature stress exposure revealed some variation among the coral cohorts, as visualized by principal component analysis (PCA, Figure 4a) and quantified through differential gene expression analysis (NCBI Sequence Read Archive under the BioProject accession number PRJNA1522549; Dataset S1A–F). The AGF Curaçao × Puerto Rico (CU × PR) cross exhibited the most differentiated transcript abundance profile, clustering distinctly from other cohorts along the first principal component (PC1), which explained 23% of the variance between cohorts. Comparisons between CU × PR and cohorts with a Floridian genetic background (CU × FL and FL × FL) yielded the highest number of differentially expressed genes (DEGs), with 3928 and 4105 DEGs found within the FL × FL cross and the CU × FL cross, respectively (Dataset S1A,B). Gene Ontological (GO) Enrichment analyses (Dataset S2A–M) showed pathways involved in lipid biosynthesis and storage, particularly sphingolipids, were consistently downregulated in CU × PR when compared to CU × FL and FL × FL, pathways related to tissue development and cell proliferation (“regulation of epithelial to mesenchymal transition”, “regulation of cell cycle process”; Dataset S2D,E).

The Curaçao (CU × CU) cross also showed significant differences in transcript abundance, with 2841 DEGs when compared to CU × PR (Dataset S1C). While less extreme than the CU × PR comparisons, the CU × CU cohort similarly diverged from Florida‐containing cohorts, with ~180 DEGs for both CU × FL and FL × FL comparisons (Dataset S1D,E), leading to a slight separation in the PCA clustering of the CU × CU and FL × FL cohorts that explained 10% of variation along the second principal component (PC2; Figure 4a; Dataset S1D,E). In contrast, CU × FL and FL × FL demonstrated the highest transcriptomic similarity, with only 8 DEGs between them (Dataset S1F). Of these eight differentially expressed genes, higher expression was found in the CU × FL cohort for the *collagen triple helix repeat‐containing protein 1* (*cthrc1*) gene as well as enrichment pathways related to wnt protein binding. The innate immune response gene *C‐type mannose receptor 2* was upregulated in the FL × FL cohort.

#### 3.5.2 Transcriptomic Response to Elevated Temperature Conditions, Within Cohorts

Of the four cohorts, only the AGF CU × FL and CU × PR showed a significant transcriptomic change at the end of the experiment after thermal stress exposure, with 175 and 81 differentially expressed genes found (respectively, Dataset S1G,H), compared to the 34 and 20 DEGs in the FL × FL and CU × CU cohorts (Dataset S1I,J). PCA analyses revealed substantially overlapping distributions of treated and ambient samples for these latter two cohorts (Figure 4c,d). The CU × CU cohort showed two subclusters that did not align with treatment assignment (Figure 4c). In contrast, samples were weakly separated based on treatment into two clusters for the CU × PR (Figure 4e) and CU × FL cohorts (Figure 4f).

Only found in the CU × PR cohort, *hsp70,* a heat shock protein, was under‐expressed (−0.58 log_2_‐fold decrease) as were other stress response genes including *cytochrome P 450 3A18*, an oxidative stress related gene (−0.94 log_2_‐fold change), an apoptosis regulator *TNF receptor‐associated factor 6* (−0.89 log_2_‐fold change), and *complement factor B*, a key player in innate immunity (−1.5 log_2_‐fold change).

Despite 81 genes being differentially expressed, the CU × FL cohort's transcriptional response at the conclusion of the experiment was similar to the ambient treatment, apart from the calcium‐modulated protein, *Calmodulin*, and *Myosin‐2 essential light chain*, both of which were slightly under‐expressed. Instead, the response to high temperature exposure was characterized by the upregulation of structural genes such as *collagen triple helix repeat‐containing protein 1* and the downregulation of metabolic and cellular process genes including *universal stress protein and thioredoxin domain‐containing protein*. Gene Ontology analysis revealed greater enrichment in terms related to tissue remodeling and morphogenesis (Dataset S2G), including “regulation of canonical Wnt signaling pathway” (GO:0060828) and “tissue remodeling” (GO:0048771).

Though showing minimal transcriptional response, the FL × FL cohort was the only other cohort besides CU x PR that significantly upregulated any heat shock proteins, specifically *hsp16* (1.97 log_2_‐fold increase), as well as enrichment pathways (Dataset S2H) related to ‘response to heat’ (GO:0009408) and ‘response to temperature stimulus’ (GO:0009266). Of the remaining 19 DEGs, the 8 annotated genes did not have any known role in heat stress response.

Notably, the majority of DEGs across cohorts were downregulated by the conclusion of the experiment, with the exception of CU × CU, which displayed a unique pattern of predominantly upregulated genes (33 vs. 1 DEG). One such gene included *Sacsin* (2.3 log_2_‐fold increase), a co‐chaperone for the heat‐shock protein Hsp70.

#### 3.5.3 Transcriptomic Response to Elevated Temperature Conditions, Between Cohorts

The magnitude of pairwise transcriptomic differences between cohorts (Dataset S1K–O) decreased after heat stress (Figure 4b), though generally followed the same trends as observed at baseline conditions. Indeed, most differences were found when comparing CU × PR to the non‐AGF CU × CU cross (162 DEGs; Dataset S1O), which included “oxidative stress‐responsive serine‐rich protein 1” (log_2_FoldChange = −1.06; *p*adj = 0.05), “Forkhead box protein L1” (log_2_FoldChange = −1.67; *p*adj = 0.024), and “metalloproteinase inhibitor 3” (log_2_FoldChange = −2.09; *p*adj = 0.003). A total of 222 differentially expressed genes were found between CU × PR and the FL × FL cohort with several transcripts showing notable upregulation (Dataset S1N). For example, ‘bacterial dynamin‐like protein’ (log_2_FoldChange = 3.86; *p*adj = 0.027) and ‘allene oxide synthase‐lipoxygenase protein’ (log_2_FoldChange = 2.54; *p*adj = 0.049) were the most overexpressed genes in the CU × PR population corals compared to FL × FL, and transcripts commonly associated with stress responses were also differentially expressed. Of note, heat shock protein ‘Hsp‐16.2’ (log_2_FoldChange = −1.75; *p*adj = 0.041) was downregulated in CU × PR relative to FL × FL corals, while ‘peroxidasin’ was upregulated (log_2_FoldChange = 1.1; *p*adj = 0.01).

The other AGF population, CU × FL had a more similar response to the non‐AGF populations, with only 8 differentially expressed genes when compared to FL × FL, and 61 differentially expressed genes compared to the CU × CU population (with only 4 genes annotated, none of which have a known role in thermal stress response; Dataset S1K,L, respectively). When comparing CU × FL to CU × CU expression profiles, “TRAF6”, which mediates cellular stress signaling and immune responses, was significantly downregulated (log_2_FC = −1.17, *p*adj = 0.041). “Cytochrome P450”, involved in oxidative stress responses, also showed decreased expression (log_2_FC = −1.79, *p*adj = 0.013). Heat shock proteins and other canonical thermal stress response genes were absent from transcriptomic responses.

Of our various transcriptomic contrasts, the AGF cohorts (CU × PR and CU × FL) had the greatest transcriptomic shifts by the end of the experiment. After heat stress, there were only two differentially expressed genes between them, neither of which is annotated (Dataset S1M). Likewise, the two non‐AGF cohorts (CU × CU and FL × FL) had similar transcriptomic profiles with only 15 differentially expressed genes between them after heat stress exposure.

**FIGURE 5:**
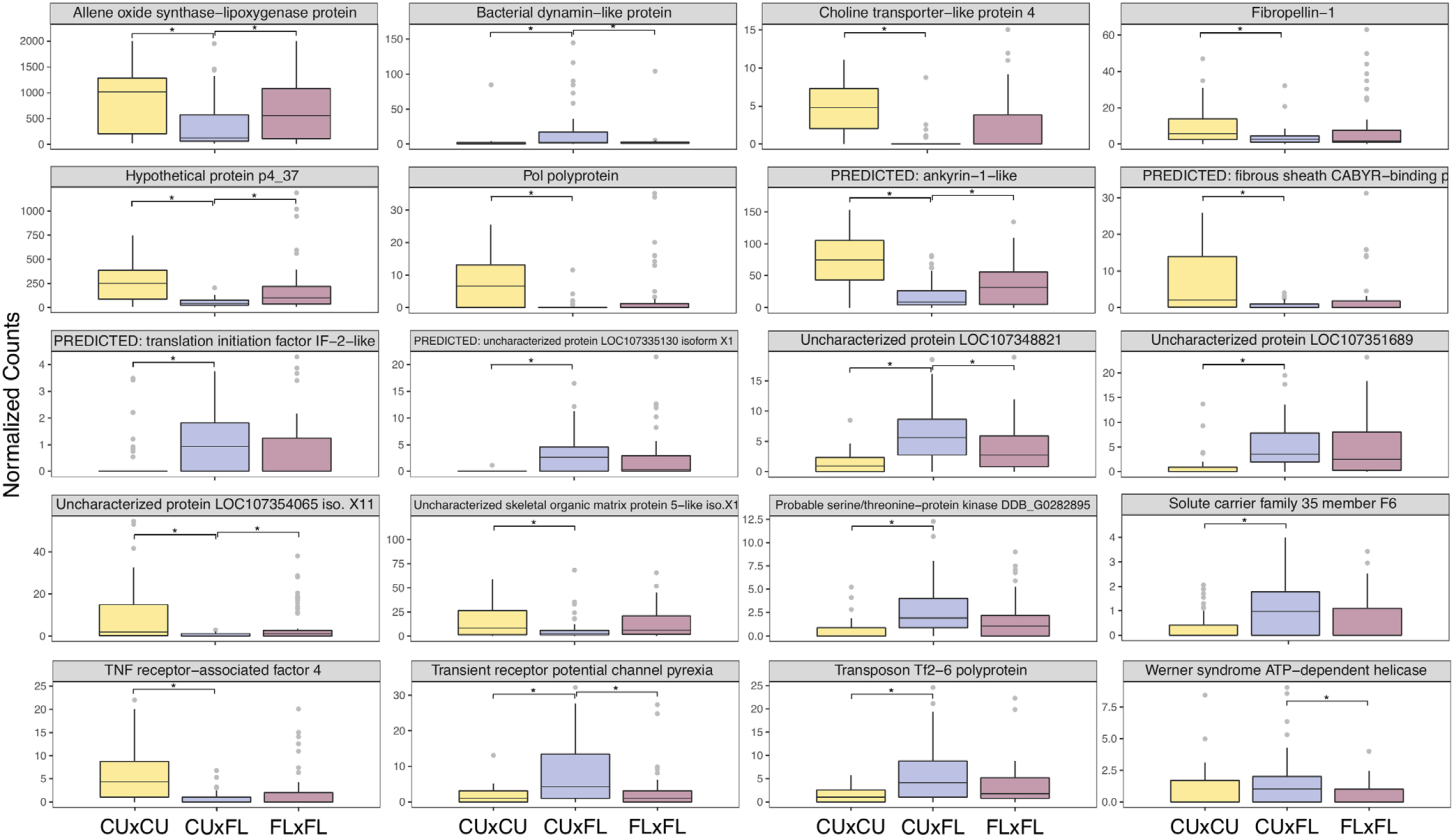
Expression differences in CU × FL cohort compared to purebred parental cohorts. Boxplots show normalized gene counts for the top 20 genes deviating most from the mid‐parent expected value in the CU × FL cohort (blue), relative to the purebred parental cohorts CU × CU (yellow) and FL × FL (pink) under pre‐exposure conditions. Asterisks indicate significant differences.

To explore mid‐parent deviation of gene expression in an AGF population (Figure 5), normalized counts of the CU × FL population were compared to the mean of purebred FL × FL and CU × CU populations, yielding 26 upregulated and 48 downregulated genes in the AGF cross (alpha = 0.05, lfcThreshold = 0.1). Pairwise comparisons showed a greater similarity between the CU × FL and FL × FL cohorts than the CU x CU cohort (with 1 DEG with the FL x FL cohort versus 20 DEG with the CU × CU cohort).

### 3.6 Symbiont Typing

The results of both a linear discriminant analysis of the signal intensities and a second random forest analysis indicate that all corals used in the experiment hosted algae in the genus *Durusdinium* (Table S3).

### 3.7 Association Analysis

Among all 19,695 SNPs, 6700 polymorphic SNPs were retained for the association analysis. Across all 5 traits tested, no significant associations were detected. The trait that had the strongest overall contribution to the variation in the data (*F*_v_/*F*_m_) was selected to plot the distribution of *p*‐values across chromosomes of the *Acropora palmata* genome (Figure S1).

### 3.8 Genetic Diversity

**FIGURE 6:**
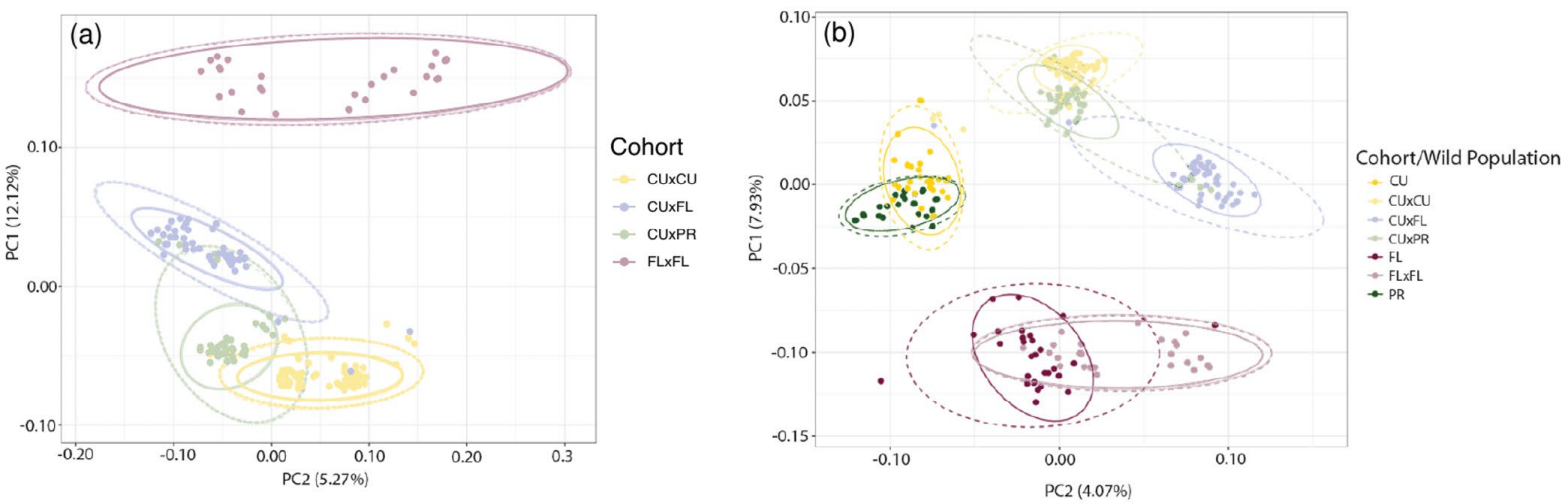
Genetic divergence of cohorts. (a) Principal Component Analysis (PCA) of pairwise genetic differences between all samples calculated using 19,695 SNPs. 12.41% of variation is explained by Principal component (PC) 1 and 5.23% of variation is explained by Principal component (PC2) 2. As expected, the FL × FL corals cluster distinctly from the remainder of the cohorts, demonstrating their unique ancestry relative to the other samples. (b) PCA of pairwise genetic differences of the four AGF cohorts and randomly selected wild genets from the three source populations (FL, CU, PR). FL wild and experimental cohorts are well differentiated from the other cohorts and populations while PR and CU wild populations are more genetically similar. CU × CU and the FL × FL crosses show shifts in allele frequencies relative to the wild population, likely due to stochasticity as well as selection imposed during larval rearing.

The principal component analysis (Figure 6a) showed that the FL x FL cohort was quite distinct from the other three cohorts (metadata available in Dataset S3). This was expected, as all three other cohorts contained some proportion of Curaçao ancestry, sharing the same five dams from Curaçao. Also, the CU × FL cohort clustered more closely to the FL × FL cohort than any other cohort. The FL × FL cohort, while being genetically distinct from the three other cohorts, also had the largest genetic variation within itself, spreading across principal component 2. This is likely because corals in the FL × FL cohort were derived from a larger number of parents (five dams and five sires). In the CU × CU cohort, two subclusters are visible in the PCA, likely reflecting shared parentage (Figure 6a), but overall genetic variation within the CU × CU cohort was small due to the limited number of total genets in the broodstock. When randomly selected wild samples were included in the principal component analysis (Figure 6b), the FL × FL cohort clustered well with the wild FL samples, while the AGF cohorts with Curaçao ancestry clustered much closer to each other rather than with the wild samples of their source populations, highlighting their unique genetic background.

**FIGURE 7:**
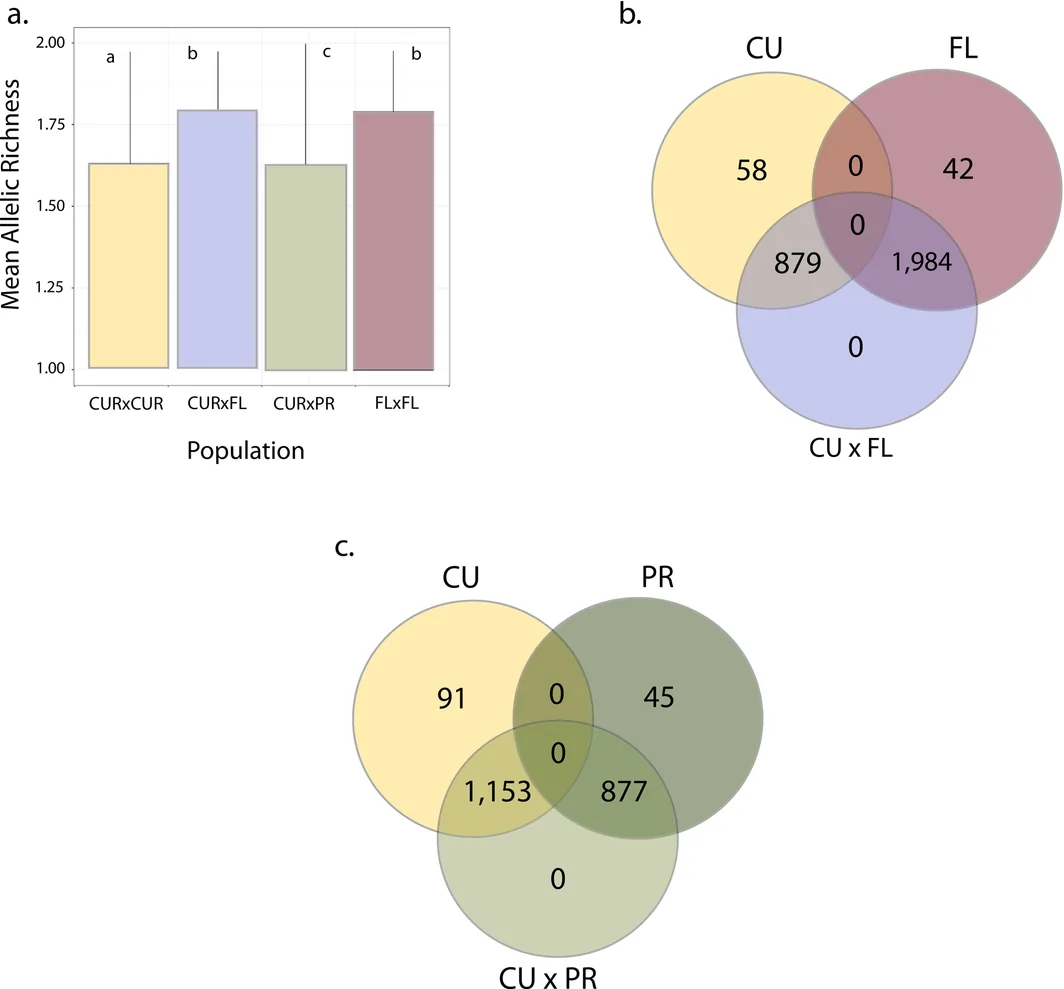
Allelic diversity and private alleles in AGF populations. (a) Mean allelic richness across all 19,695 loci. There were significant differences (indicated by lower letters) in allelic diversity across most cohorts. The FL × FL cohort shows the greatest mean allelic richness. However, there was little reduction in genetic diversity due to the smaller number of parents used to create the cohorts of Curaçao ancestry (b) Venn Diagram of private alleles shared in the CU × FL and (c) CU × PR cohorts. The CU × FL cohort contained 879 alleles from Curaçao not previously recovered in the Florida populations, and the CU × PR cohort contained 1153 alleles from Curaçao not previously recovered in the Western Puerto Rico populations.

There were significant differences (*p* < 0.05) in mean allelic diversity between all cohorts (Figure 7a). While the mean allelic richness was significantly different between the CU × CU and CU × PR cohorts, CU × FL and FL × FL cohorts (Figure 7a), it is important to note that the range of mean allelic richness of all cohorts was small [1.236 (CU × CU) to 1.299 (FL × FL)]. Between CU and FL wild populations, there were 2963 private alleles, 2026 of which were private to FL and 937 of which were private to CU. The CU × FL AGF cohort contained 879 private CU alleles and 1984 private FL alleles (Figure 7b). Between CU and PR wild populations, there were 2166 private alleles, 922 of which were private to PR and 1244 of which were private to CU. The CU × PR cohort contained 1153 private CU alleles and 877 private PR alleles (Figure 7c).

Pairwise kinship coefficients were calculated between all corals in the experiment and analyzed within and between cohorts with an ANOVA. A mean kinship above 0 indicates that recruits are highly related. The CU × FL cohort had the highest mean kinship of 0.118 (second degree relationship) within its cohort, while the FL × FL population had the lowest mean kinship within its cohort of −0.0360 (unrelated). Overall, the FL × FL cohort contained more unrelated recruits. This reflects the results from the PCA, showing that the FL × FL cohort is not only genetically distinct from the other cohorts as previously stated, but also has lower within‐cohort kinship due to the larger number of parents contributing.

Average observed heterozygosity of the CU × CU cohort was 0.136 ± 0.022. Average observed heterozygosity of the CU × FL cohort 0.135 ± 0.021. Average heterozygosity of the FL × FL cohort was 0.149 ± 0.024. Average heterozygosity of the CU × PR cohort was 0.138 ± 0.022. None of the cohorts significantly differed from expected heterozygosity. The CU × CU and CU × PR cohorts had slightly lower *F*_IS_ values, while the CU × FL and FL × FL cohorts had slightly higher *F*_IS_ (Table 2). These values are within the expected range as detected in wild populations of *A. palmata* using the same genotyping method (Kitchen et al. 2020).

**TABLE 2:** Heterozygosity of cohorts measured in the follow‐up heat stress study. Observed and expected heterozygosity were calculated for each cohort examined in this study, along with F_IS_ (inbreeding coefficient). No significant deviation from Hardy–Weinberg expectations was found.

Cohort	N_Loci	Observed heterozygosity	Expected heterozygosity	*F*_IS_
CU × CU	19,695	0.136	0.138	0.0098
CU × FL	19,695	0.135	0.147	0.0797
CU × PR	19,695	0.139	0.137	0.0126
FL × FL	19,695	0.15	0.14	0.0726

## 4 Discussion

In this study we present the first comparative dataset characterizing the physiology, gene expression, and genetic diversity of F1 crosses from purebred and AGF cohorts of the critically endangered reef‐building coral, *Acropora palmata* . The results showed little physiological differences between AGF and purebred cohorts regardless of whether they were under ambient or high temperature conditions, although purebred cohorts grew significantly faster than the AGF cohorts. Additionally, the AGF cohorts included novel alleles and unique gene expression profiles not found in Florida, which could provide conservation value. These data indicate the F1 AGF cohorts created from Hagedorn et al. (2021) may be good candidates for further assessing the integration of AGF for genetic rescue purposes in Florida.

### 4.1 AGF Cohorts Were Physiologically Similar to Purebred Cohorts

There were no differences detected among cohorts for most physiological metrics analyzed individually (algal symbiont and metabolism metrics) as well as when a suite of these metrics was compared using multivariate statistics. The efficacy of AGF provenancing strategies has been evaluated across several coral species in Australia, where significant differences in survival, growth, and heat tolerance were detected among cohorts, with outcomes varying across life stages (Macadam et al. 2025). One possible explanation for the absence of physiological differences among cohorts under both ambient and elevated temperature conditions in the present study is that the parental populations were selected primarily as a proof‐of‐concept to demonstrate the feasibility of cryopreservation for genetic rescue of *A. palmata* in Florida, rather than being strategically chosen along an environmental gradient to maximize climate adaptation potential. However, Quigley et al. (2021) demonstrated that even when strategic provenancing is integrated, many AGF cohorts may be similar to the purebred ‘local’ cohorts. These explorations into the efficacy of AGF are the first steps of many to understand the utility of this tool for population management of these threatened species.

### 4.2 Purebred Cohorts Showed Significantly Higher Growth Rates When Compared With the AGF Cohorts

Interestingly, the FL × FL purebred cohort showed significantly faster 2DSA growth rates when compared with the other cohorts. Additionally, the CU × CU purebred cohort showed significantly faster 3DSA growth rates when compared with the CU × FL and CU × PR AGF cohorts. A recent study assessing the survival and growth rates of corals created using AGF in Australia also showed significantly reduced growth rates of AGF cohorts compared with control crosses of *Acropora tersa* (Macadam et al. 2026). The Macadam et al. (2026) study also showed that AGF corals had enhanced survival during high temperature conditions compared with controls, indicating the potential utility of AGF for promoting thermal tolerance within coral populations, but it could also underscore potential tradeoffs between heat tolerance and growth. Another aspect that could have affected the growth rates of the AGF cohorts within the present study was the process of sperm cryopreservation utilized to create these cross‐jurisdictional coral recruits. Cryopreserved sperm was utilized for both the CU × FL and the CU × PR AGF crosses, but only fresh sperm was utilized for the purebred crosses. This approach mimics real‐world scenarios for implementing AGF within the Caribbean region, where live batches of sexually created cohorts are standard practice within jurisdictions, but cryopreservation is the most feasible way of conducting AGF between jurisdictions due to permitting and logistical constraints. Although cryopreservation reduces sperm survival and motility, thawed sperm that are highly motile can successfully fertilize eggs with no evidence of adverse effects on coral physiology during settlement or growout (Hagedorn et al. 2017).

### 4.3 High Temperature Exposure Did Not Reach the Corals' Thermal Threshold

Although the cohorts were exposed to water temperatures that caused extensive bleaching of *A. palmata* under natural reef conditions of the Florida Keys several times in the past decade (Manzello et al. 2007; Williams et al. 2017), the corals within the present experiment showed no signs of physiological stress associated with exposure to 31°C for 2 months. Eight weeks at 31°C is equivalent to 11.2 DHW in Florida (MMM = 29.6°C), which is predicted to lead to significant bleaching and mortality. The most recent and extremely severe, widespread bleaching event occurred in 2023 with water temperatures exceeding 20 DHWs, which led to almost complete loss of *A. palmata* throughout Florida (Manzello et al. 2025). However, all previously recorded bleaching events from 1985 to 2022 had water temperatures that resulted in less than 10 DHWs (Neely et al. 2024), which still caused extensive mortality for Aspeicroporids on reefs in Florida with no signs of acclimation (Williams et al. 2017). Within this context, the 11.2 DHW the *A. palmata* were exposed to in the present study should have led to significant bleaching. However, the typical algal symbionts within wild populations were likely *Symbiodinium ‘fitti’* (Baums et al. 2014), which is more thermally sensitive than those measured within the present study's experimental corals.

### 4.4 The Dominant Algal Symbiont, *Durusdinium trenchii*, Likely Influenced Phenotypic Response

The lack of a negative stress response associated with the high temperature exposures from the cohorts may be from the unique association between the *A. palmata* reared within the study and the dominant algal symbiont identified within each of the cohorts, *Durusdinium trenchii*. This particular algal symbiont is significantly more heat tolerant than other species of Symbiodiniaceae (LaJeunesse et al. 2014; Manzello et al. 2019), which may have provided thermal protection to the extended high temperature conditions within the study. Indeed, most *A. palmata* in situ are associated with a more temperature sensitive algal symbiont, *Symbiodinium* ‘*fitti’* (Baums et al. 2014; Durante et al. 2019) and typically experience thermal stress starting at 30.5°C in the Upper Florida Keys, with high mortality occurring when temperatures exceed 31.3°C (Williams et al. 2017). These results indicate that the experimental corals used within the present study may be more thermally tolerant than the naturally occurring populations because of the unique host/algal symbiont relationship, which developed as an artifact of rearing location, Mote's land based coral nursery. Whether these more heat tolerant symbionts are retained after outplanting is not well known. Elder et al. (2023) showed that Mote‐sourced *A. palmata* corals outplanted with *D. trenchii* retained that algal symbiont for up to 2 years. These results suggest that the juvenile hosts and the novel symbiont may maintain their association for several years post introduction to the natural reef environment even if the host eventually reverts to the analogous symbiont, *S. ‘fitti’*. On the contrary, Gantt et al. (2025) showed an almost immediate shuffling from *D. trenchii* to *S*. ‘*fitti*’ of Mote‐sourced *A. palmata* corals, evident within just one year after outplanting. Indeed, further study is needed to fully understand the influence of this novel host‐symbiont association and the persistence of the association, or not, through time.

### 4.5 High Water Temperatures Increased Growth Rates

Not only was there no significant negative effect of high‐water temperatures on any cohort, but there was also even a statistically higher two‐dimensional growth rate for all the cohorts under these high temperature conditions. All cohorts even showed a trend of higher growth rates (2DSA, 3DSA, and BW) under the high temperature treatment, some of which were statistically significant (e.g., CU × PR 2DSA). Previous studies suggest a Gaussian distribution occurs between water temperatures and calcification rates where a positive correlation takes place until a thermal threshold is reached, the symbiosis between the coral host and the algal symbiont breaks down, bleaching occurs, and growth rates decline (Jokiel et al. 1978). Since there were no negative physiological consequences of a two‐month exposure to 31°C within the study, this suggests that the corals did not reach their thermal maximum and were rapidly growing two dimensionally throughout the study.

### 4.6 CU × FL Cohorts Contain Novel Allelic Diversity Not Known to Be Present in the FL Population

One of the benefits of AGF is the introduction of novel alleles to a declining population (Frankham et al. 2017). Standing genetic variation is important to provide the material for future adaptation (Barrett and Schluter 2008). Florida acroporids have suffered catastrophic declines and only ~150 founder genets currently exist (Williams et al. 2024). The AGF crosses carry > 1000 alleles that are not currently known to be present in the Florida population according to the assay of 19,696 loci, and as such present a valuable source of novel alleles and means to increase standing genetic variation within Florida, which is not achievable by pure‐bred crosses. The 19,696 SNP loci genotyping array indicated that 879 novel alleles could be introduced from Curaçao into the Florida population alone. The CU × FL AGF cohort may thus provide substantial additional genetic diversity to avoid inbreeding and allow a declining FL population to adapt to changing environmental conditions. While the Curaçao wild population is much more genetically similar to Western Puerto Rico (Figure 6a) compared to the other populations, there were still 1153 private Curaçao loci that could be potentially introduced as adaptive standing genetic variation into Puerto Rico populations.

The genetic data also highlighted that coral cohorts created from a limited number of parents are necessarily closely related (Baums et al. 2022). Therefore, when employing AGF, care must be taken during outplanting of offspring to reduce the risk of future inbreeding between siblings. This is best achieved by limiting the number of related recruits that are placed near each other on a reef. Recent data from the Pacific (Mumby et al. 2024) indicated that in that system, over 10 m should be effective in severely reducing fertilization opportunities.

### 4.7 AGF Cohorts Provide Novel Differential Gene Expression and Parallel Phenotype

Gene expression analyses on corals created through assisted gene flow have rarely been conducted before. Limited work from other regions shows that inter‐population crossing can produce offspring with enhanced thermal tolerance. For example, crosses within and between *Acropora millepora* parents from a high and a low latitude population exhibited heritable differences in gene expression and phenotype under thermal stress (Dixon et al. 2015). Similarly, when crossing *Platygyra daedalea* mothers from relatively cool regions in the Indian Ocean with fathers from hot reefs in the Persian Gulf (Howells et al. 2021), these AGF created offspring had a 37% increase in heat survival over purebreds from the cold location. Across both studies, variation in stress tolerance among AGF cohorts exceeded what would be predicted from population origin alone, indicating that individual‐level genetic variation among parents, alongside any broader population‐level effects, can significantly influence AGF cohort performance.

In line with these results, we found that baseline transcriptomic profiles diverged across the four cohorts even before thermal stress was applied, with CU × PR showing the most distinct profile and CU × FL most closely resembling FL × FL. Among the few baseline DEGs distinguishing CU × FL from FL × FL, *cthrc1*, a secreted glycoprotein previously linked to tip growth in *Acropora* (Hemond et al. 2014), was upregulated in CU × FL, suggesting that AGF crossing produced constitutive differences in growth‐related transcriptional programs that were present from the outset.

These constitutive differences gave way to divergent response strategies under thermal stress. The CU × PR cohort showed downregulation of canonical stress‐response genes typically upregulated in coral thermal stress, including *hsp70*, *cyp450*, *traf6*, and *cfb*, suggesting a suppressed or potentially maladaptive response. CU × CU was distinctive in showing predominantly upregulated genes (33 of 34 DEGs), including the Hsp70 co‐chaperone *Sacsin* (Fuller et al. 2020), while FL × FL was the only cohort to upregulate canonical heat‐shock genes (*hsp16*) and enrich for “response to heat” GO terms. CU × FL diverged from all three. Rather than canonical heat‐shock genetic responses, this cohort enriched for tissue remodeling and Wnt signaling pathways alongside continued upregulation of *cthrc1*, suggesting a response based on tissue maintenance and structural pathways. One possible explanation is that the 879 private Curaçao alleles introduced into the CU × FL cohort contribute to standing regulatory variation in stress‐response pathways, with effects that may be subtle, context‐dependent, or distributed across many loci rather than producing strong gene‐level differential expression.

Beyond these cohort‐level response patterns, the mid‐parent deviation analysis identified regulatory novelty in the AGF cohorts. Seventy‐four genes (26 upregulated, 48 downregulated) showed CU × FL baseline expression that diverged from the additive expectation calculated from FL × FL and CU × CU. Such deviations are categorized in hybrid biology as non‐additive inheritance, including parental dominance, over‐ or under‐dominance, and transgressive expression (Yoo et al. 2014), and represent regulatory phenotypes that cannot be predicted from parental averaging. Several of these deviating genes are homologs with characterized roles in coral biology, including *allene oxide synthase‐lipoxygenase*, active in *Acropora* biosynthesis and linked to thermal stress in other corals (Lõhelaid and Samel 2018), and a homolog of *skeletal organic matrix protein*, central to *Acropora* biomineralization (Ramos‐Silva et al. 2013). Whether this baseline regulatory novelty translates to differences under stress remains to be tested, but it is a form of variation that purebred Florida crosses, on their own, cannot generate and may provide valuable information for managers to consider the value of the integration of AGF cohorts into Florida reefs.

4.8 | Looking Ahead AGF corals have not yet been outplanted into Florida's Coral Reef, but this may be a possibility soon. The result of the present study suggests that the specific AGF cohorts created by Hagedorn et al. (2021) have phenotypes similar to native purebred populations, may harbor flexibility in symbiont associations, and would introduce novel alleles and gene expression profiles not observed in either purebred cross, representing potentially adaptive variation. This reinforces the conservation value of AGF corals, particularly CU × FL, as a source of alleles and new molecular phenotypes that may enhance resilience in Florida's declining coral population. Next steps to assess feasibility for targeted integration include field trials for the existing AGF cohorts for direct comparison with the local population and backcrossing the F1 CU × FL cohort with FL × FL purebreds to assess the risk of outbreeding depression. Incorporating AGF into large‐scale population recovery efforts will require careful planning that accounts for both the asexual and sexual reproductive potential of the species. However, data generated from the present study, alongside future field trials, would provide the foundation necessary to parameterize population models and inform such recovery strategies.

## 5 Conclusion

This work demonstrates the potential power of assisted gene flow when conducting active restoration for the purpose of species conservation. Perpetual poor performance of local populations in recent years, e.g., *A. palmata* in Florida's Coral Reef, may suggest that whatever local adaptation is present in these populations is insufficient to ensure persistence as environmental conditions continue to change and decline (Williams et al. 2008, 2014; Chan et al. 2019; Baums et al. 2019; Manzello et al. 2025). Thus, the effective population size may be so degraded that inbreeding depression is of increasing concern. In contrast, the genetic risks associated with assisted gene flow (e.g., outbreeding depression) may be nominal. The present study showed high levels of phenotypic similarity among the four cohorts tested even after exposure to elevated temperatures for two months and despite the varied number of parents contributing to each. The novel relationship between the coral host and *Durusdinium trenchii*, a resulting phenomenon associated with reared *Acropora* spp. within Mote's land‐based coral nursery, suggests all these cohorts may be more resistant to thermal stress compared with those reared within the wild. Additionally, CU × FL individuals harbor genetic diversity not previously found in Florida that could be potentially adaptive. An assisted gene flow strategy utilizing distantly located populations can therefore promote the transfer of adaptive variation into genetically‐depauperate populations as a means of genetic rescue. However, depending on the mechanism(s) underlying outbreeding depression, fitness reductions may be observed within F1 generations, but perhaps not until after the F2 generation (Willis et al. 2006; Baums 2008). Therefore, to understand the long‐term impacts of AGF on coral fitness, further research is necessary, which could include controlled breeding studies and common garden experiments. As Florida's *A. palmata* has now reached functional extinction, this study provides the initial scientific groundwork urgently needed to deploy AGF as a viable, and perhaps essential, population management strategy.

## Funding

This work was supported by the Paul G. Allen Family Foundation (Request #12894). While working on this project, E.M.M. was supported by a Mote Eminent Scholarship, I.B.B. was supported by a NOAA grant NA20NMF4820320. C.C.O. was supported by an NSF‐GRFP (#2020289929), T.C. was supported by a PSU Eberly College SAGF fellowship. Mote Postdoctoral Research Fellowship and German Research Foundation (DFG) (394448490).

## Conflicts of Interest

The authors declare no conflicts of interest.

## Supporting information


**Figure S1:** Manhattan Plot of Genome‐wide *p*‐values for SNPs associated with variation in *F*_v_/*F*_m_ in cohorts tested in this study.
**Table S1:** Phenotype data. A suite of physiological parameters measured to assess the phenotypes of the different cohorts under the ambient and high temperature conditions.
**Table S2:** The subset of phenotype data used in the multivariate analysis that has been normalized through log transformation.
**Table S3:** Detected Symbiodiniaceae in *A. palmata* tissue samples.
**Dataset: S1** Differential gene expression (DEG) results from baseline and heat stress contrasts.
**Dataset: S2** Functional enrichment of significant DEGs from baseline (ambient condition) and heat stress contrasts.
**Dataset: S3** Associated metadata of the SNP arrays to access the raw CEL files used to generate genotyping calls for population genetic analyses of *Acropora palmata* samples.

## Data Availability

The data that supports the findings of this study are available in the Supporting Information of this article.
